# Supplementary material for: The Effect of Providing Staff Training and Enhanced Support to Care Homes on Care Processes, Safety Climate and Avoidable Harms: Evaluation of a Care Home Quality Improvement Programme in England
Source: Int J Environ Res Public Health. 2021 Jul 16;18(14):7581. doi: 10.3390/ijerph18147581 (PMC8307011; doi:10.3390/ijerph18147581)
Supplement: Supplementary file 1 [file ijerph-18-07581-s001.zip › ijerph-1284755-supplementary.pdf]

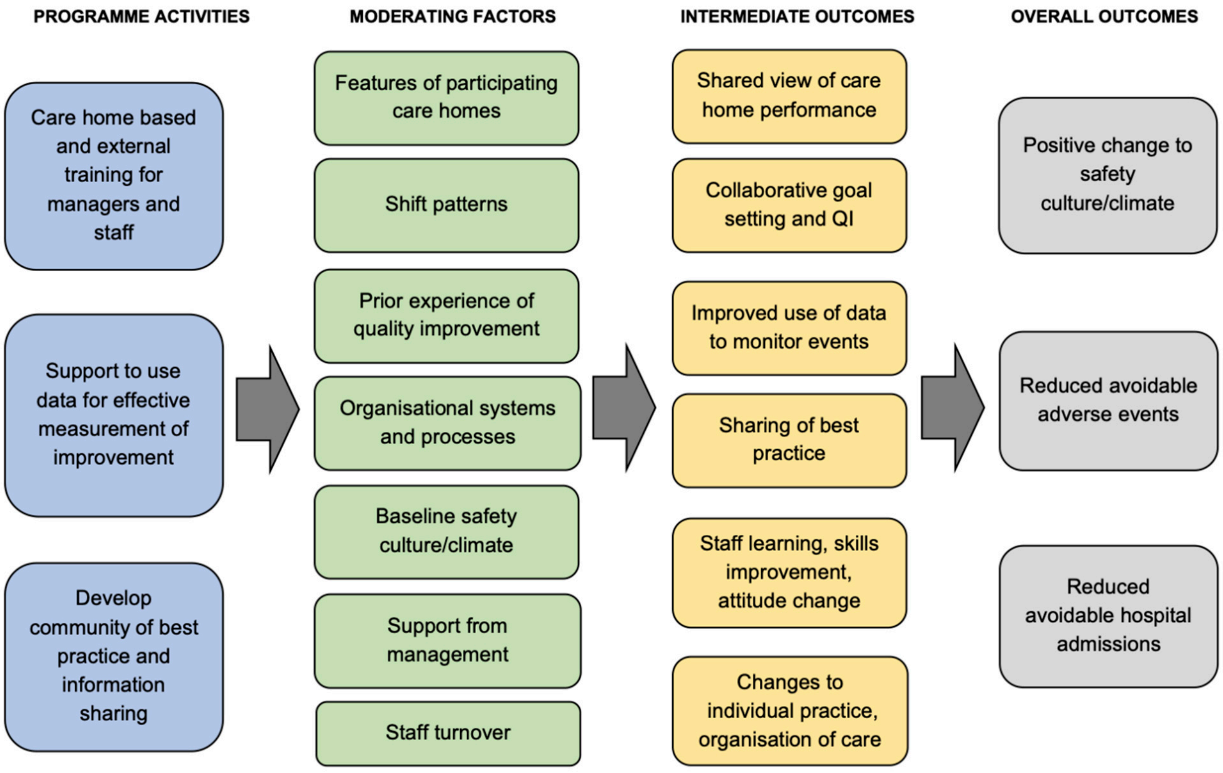

Figure S1. Theory of change underpinning SPACE programme.

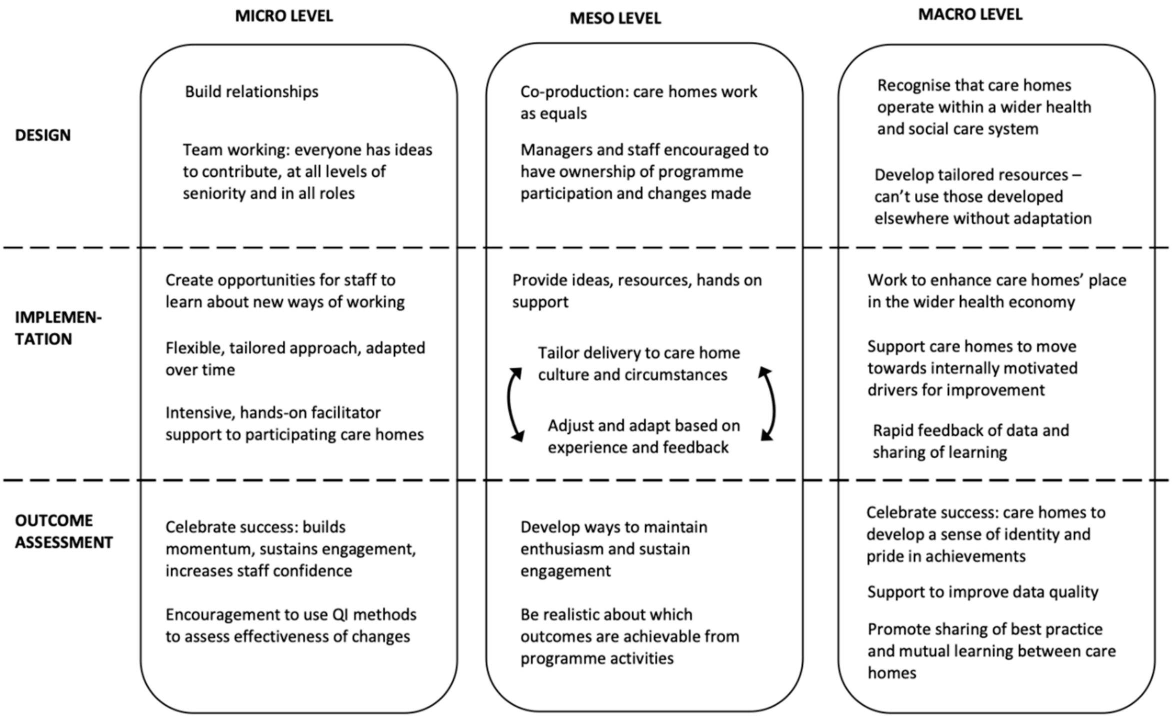

Figure S2. Mapping SPACE success factors across system levels.

Table S1. Quotations from semi-structured interviews.

No.	Quotation and Role of Participant

1	*"The falls training really impacted me because since I've had that, I'm changing the way I'm looking at someone…and I'm coming up with new ideas"* (Care assistant)
2	*"Safety isn't just falls, it's the whole bigger picture, isn't it? So I was just starting to look at the bigger picture rather than just what led up to the fall"* (Care home manager)
3	*"It's a day to day thing you see…we sit and suggest amongst ourselves what we could do…If it works, carry on, if it doesn't, then think of something else"* (Care assistant)
4	*"People are on board and people are coming up with* [ideas] *and it just becomes the way…we're flying!"* (Senior care assistant)
5	*"Staff are a lot more switched on and they're enjoying it because they've got that responsibility"* (Care assistant)
6	*"They're looking at the data themselves…they're thinking about their trends…they are grasping that now and owning their data"* (SPACE facilitator)
7	*"It's a learning process every day, even through my knowledge and my experience, it's good to have a bit of background and that bit more knowledge, for us to carry it out and pass it on to other colleagues"* (Care assistant)
8	*"Sharing information is a massive part and it's very much created that team feeling…normally you have homes against each other because it's a fight for beds…Because of this programme we're all now a team"* (Care home unit manager)
9	*"Since SPACE I did get more confidence from it because people were listening to me. I'm confident in my knowledge, I'm confident with the support"* (Senior care assistant)
10	*"The first year was looking at raising the profile of quality improvement and then looking at the fundamentals of pressure injury, falls, nutrition and hydration…but staff have evolved from that and run with it and are doing their own things"* (SPACE facilitator)
11	[Care home manager] *is not very IT savvy…so sitting down with him, sending him his monthly trends out, he's like 'Hmm' and starting to look at them"* (SPACE facilitator)
12	*"We always concentrate on the negatives—the reds and the ambers, you know…You're not used to looking at the greens and thinking about what went right and how can that be replicated?"* (SPACE facilitator)
13	*"It's not us doing it to them, we're doing it with them and they're influencing in terms of where their developments should be"* (SPACE facilitator)
14	"I've built a really good relationship with a lot of the managers and they're always phoning me, 'oh we're doing this, we're doing that'" (SPACE facilitator)
15	*"I felt as if they were interfering with what we were doing here and some of the ideas were so basic that it was a bit of an insult to have someone come in and say 'you should be doing this and you should be doing that'…from my point of view it was annoying, but to see the enthusiasm that she and the programme developed with the care staff was great!"* (Care home deputy manager)
16	[SPACE has impacted] *"Massively, you know, we've got the ideas and everything, but without the help of SPACE, with all the different projects going on, and the support, that's why it's been so successful"* (Care home manager)
17	*"It's easy to understand. It's accessible. We can put up trends for the staff and say 'look how fantastically we're doing with our falls, look how brilliantly we're doing with pressure injuries…we've had a few grade 2s but that's good because we're picking them up early"* (Senior care assistant)
18	[Managers and staff] *"are starting to see that quality is fundamentally part of their job"* (SPACE facilitator)
19	[We have had negative experiences with] *"quality improvement teams who don't understand the whole thing about nursing homes and I was sort of a bit negative. Obviously* [facilitator]*'s attitude was completely different and we were glad of the help as well"* (Care home manager)
20	*"It has been brilliant actually. The SPACE project I think kind of lighted that fire in us to do things because I think you can become stagnant in the home and you carry on with your normal routine, but having those meetings and coming up with ideas it's like a buzz, you are excited to do new things, new projects, new protocols"* (Care home unit manager)
21	*"It's embedded with all the extra from SPACE. We're already for our ideas for carrying on over and above"* (Care home manager)
22	[SPACE has become] *"the new normal if you like. I feel SPACE belongs to them, they're more passionate about it"* (Care home manager)
23	*"We're so excited for it and we're so motivated…we don't want the momentum to just go, which I think unfortunately, because of the culture of care homes it will just fade, which will be really sad"* (Care home unit manager)

24	*"From the moment* [facilitator] *left her post, the emails stopped coming through about different incentives, you know, for things for us to do and courses. We barely hear from the CCG now in that respect, so disappointing, very disappointing…And I don't think we'll be represented any more like we used to"* (Care home manager)

Table S2. Comparison of mean SAQ safety climate scores between baseline and 24 months, by care home size and quality.

Statement	Variable	Grouping	Baseline [1]	24 Months	Comparison [2]
I would feel safe if I lived at this care home	Care home size [3]	Small	82.9	90.2	$p = 0.085$
		Medium	85.1	89.0	$p = 0.061$
		Large	82.2	79.9	$p = 0.387$
	Care home quality [2]	Worse	80.6	79.5	$p = 0.712$
		Better	84.4	90.1	$p = 0.0008$
Medical areas are handled appropriately	Care home size	Small	90.2	93.1	$p = 0.394$
		Medium	90.0	92.9	$p = 0.076$
		Large	87.4	86.5	$p = 0.841$
	Care home quality	Worse	88.1	87.6	$p = 0.842$
		Better	88.8	92.7	$p = 0.006$
I know who to ask about resident safety	Care home size	Small	96.4	94.1	$p = 0.302$
		Medium	93.3	94.7	$p = 0.352$
		Large	91.4	89.9	$p = 0.433$
	Care home quality	Worse	91.0	90.3	$p = 0.768$
		Better	93.8	94.7	$p = 0.439$
I receive appropriate feedback about my performance	Care home size	Small	84.8	91.7	$p = 0.068$
		Medium	82.8	81.8	$p = 0.676$
		Large	80.3	76.5	$p = 0.191$
	Care home quality	Worse	75.5	72.5	$p = 0.382$
		Better	84.3	85.9	$p = 0.373$
It is difficult to discuss errors in this care home [5]	Care home size	Small	79.8	78.0	$p = 0.770$
		Medium	65.3	73.1	$p = 0.067$
		Large	69.0	58.3	$p = 0.004$
	Care home quality	Worse	62.1	60.8	$p = 0.749$
		Better	72.2	73.4	$p = 0.643$
My colleagues encourage me to report any resident safety concerns	Care home size	Small	90.4	91.8	$p = 0.718$
		Medium	88.0	89.4	$p = 0.476$
		Large	85.0	85.8	$p = 0.754$
	Care home quality	Worse	84.7	83.8	$p = 0.767$
		Better	87.5	91.2	$p = 0.017$
The culture here makes it easy to learn from the mistakes of others	Care home size	Small	79.0	92.4	$p = 0.005$
		Medium	80.0	85.0	$p = 0.031$
		Large	82.8	83.0	$p = 0.630$
	Care home quality	Worse	79.0	80.3	$p = 0.686$
		Better	81.4	87.9	$p = 0.0004$
Aggregate mean score for domain	Care home size	Small	86.2	90.2	$p = 0.121$
		Medium	83.3	86.5	$p = 0.030$
		Large	82.7	80.90	$p = 0.149$
	Care home quality	Worse	80.1	79.3	$p = 0.704$
		Better	84.6	88.0	$p = 0.003$

[1] Higher scores = greater agreement with statement; [2] Comparisons used independent t-tests; [3] Small = <30 beds, Medium = 30-49 beds, Large = 50+ beds; [4] Care home quality categorised using the Care Quality Commission (CQC) overall rating for each care home at baseline. 'Worse' = Requires improvement/Inadequate, 'Better' = Outstanding/Good; [5] Statement is reverse scored.
